# Exploring the Composition of Protein-Ligand Binding Sites on a Large Scale

**DOI:** 10.1371/journal.pcbi.1003321

**Published:** 2013-11-21

**Authors:** Nickolay A. Khazanov, Heather A. Carlson

**Affiliations:** 1 Bioinformatics Graduate Program, University of Michigan, Ann Arbor, Michigan, United States of America; 2 Department of Medicinal Chemistry, University of Michigan, Ann Arbor, Michigan, United States of America; University College London, United Kingdom

## Abstract

The residue composition of a ligand binding site determines the interactions available for diffusion-mediated ligand binding, and understanding general composition of these sites is of great importance if we are to gain insight into the functional diversity of the proteome. Many structure-based drug design methods utilize such heuristic information for improving prediction or characterization of ligand-binding sites in proteins of unknown function. The Binding MOAD database if one of the largest curated sets of protein-ligand complexes, and provides a source of diverse, high-quality data for establishing general trends of residue composition from currently available protein structures. We present an analysis of 3,295 non-redundant proteins with 9,114 non-redundant binding sites to identify residues over-represented in binding regions versus the rest of the protein surface. The Binding MOAD database delineates biologically-relevant “valid” ligands from “invalid” small-molecule ligands bound to the protein. Invalids are present in the crystallization medium and serve no known biological function. Contacts are found to differ between these classes of ligands, indicating that residue composition of biologically relevant binding sites is distinct not only from the rest of the protein surface, but also from surface regions capable of opportunistic binding of non-functional small molecules. To confirm these trends, we perform a rigorous analysis of the variation of residue propensity with respect to the size of the dataset and the content bias inherent in structure sets obtained from a large protein structure database. The optimal size of the dataset for establishing general trends of residue propensities, as well as strategies for assessing the significance of such trends, are suggested for future studies of binding-site composition.

## Author Summary

Describing the general structure of protein binding sites is fundamentally important for guiding drug design and better understanding structure-function relationships. Here, we analyze small molecules bound to proteins within our large database, Binding MOAD (Mother of All Databases, pronounced like “mode” as a pun referring to ligand-binding modes). We focus on different contacts across the residues in the binding sites, and we normalize the data relative to the protein's entire surface. A key feature of this study is the use of a “control” where we compare real, functional binding sites to the random contacts seen for crystallographic additives against the protein surface. Controls are required in experimental biology, but they are ill-defined in many computational approaches. This allows us to describe how true binding sites are unique on the protein surface and distinct from random patches that attract common, small molecules.

## Introduction

Understanding general properties of protein-ligand binding sites is of great importance to gain insight into the functional diversity of the proteome. One of the most fundamental properties of the receptor surface is the set of amino acids available for interactions with ligands. In many protein families, this set is well known and structurally conserved due to the functional role of the residues, and several insightful studies have summarized catalytic residue content in sets of enzymes [1]–[3]. These provided insightful heuristics for predicting enzymatic sites, but the studies did not provide as much detail on non-catalytic interactions. Non-catalytic contacts cannot be ignored because they are an important component of a valid binding site, helping the ligand maintain the correct binding mode and often dictating binding specificity. Overall, the more general trend of amino-acid distribution within binding sites across a variety of protein and ligand types is less understood; previous studies have explored limited sets of proteins [4] or interactions of specific interest [5]. With ever-increasing numbers of protein structures available and numerous databases dedicated to protein-ligand analysis [6]–[10], a wider view of the residue composition of binding sites is now possible and necessary. Establishing general trends of binding-site composition can help develop valuable tools for identifying a protein functional site without prior information about the protein's structural homology. Such tools can be invaluable for the characterization of proteins emerging from current structural genomics projects [11]. The recent use of binding-site composition to bolster methods for *de novo* prediction of binding sites [12]–[15] is an encouraging example of the utility of the general binding-site composition trends.

To study the composition of ligand binding sites across the broadest set of available protein structures, we analyzed the propensity of residues in all the binding sites present in the Binding MOAD database - one of the largest sets of curated protein-ligand complexes [8]. Of course, the diversity of the database is limited to the diversity in the PDB, so there is a heavy bias toward enzyme structures and against membrane-bound systems. Also, the poor resolution of very large complexes excludes most of them from this study. Our analysis summarizes surface composition of binding sites of biologically relevant ligands, such as substrates, products, drugs, and co-factors. We also show how composition of binding-site surfaces varies with number of structures analyzed; this measure of statistical significance is not presented to this extent in other studies to date. Another unique aspect of this study is our examination of the binding of spurious co-crystals, such as crystallization buffers, solvents, and stray ions, which exhibits some markedly different trends than the binding of functional ligands.

## Methods

### Large, Non-redundant Data Set of Binding Sites

We began by assembling a non-redundant set of 3295 protein-ligand structures, each representing a closely related protein family from the 2009 release of Binding MOAD. The non-redundant set of Binding MOAD is composed of families grouped by 90% sequence identity; the 3295 complexes embody the variation of the full set of 14,720 complexes with 41,721 binding sites. A binding site was defined as the set of protein residues which have at least one non-hydrogen atom within 4.0 Å of a ligand's non-hydrogen atom. These residue interactions were then labeled as side chain (SC) or backbone-only (BB-only) depending upon which atoms participated in the interaction. A residue classified with a BB-only interaction did not have any side-chain atoms within the interaction distance. Residues were classified as SC if the interaction was solely through the side chain or through both its side chain and backbone atoms. Glycine residues are considered a special case, and interactions with glycine's Cα are always classified as SC regardless of the absence of a side chain. A single protein residue could have interactions with more than one ligand, in which case the residue interactions were considered independent, and the residue was included in each ligand's binding site provided it was within 4.0 Å. Since a ligand-based definition of the binding site was used, smaller ligands may not make contacts with all possible residues in a large binding site. Only the residues in contact with the ligand are part of the calculation of a site's solvent-accessible surface area (SASA).

In accordance with Binding MOAD annotation, each binding event is classified as “valid” or “invalid” depending on the biological relevance of the ligand [8]. Since all structures in Binding MOAD must contain a valid ligand, the likelihood of an invalid ligand occupying a biologically relevant site is greatly reduced. While it is still possible, the rate of such occurrence is much less than using all the structures in the Protein Data Bank (PDB) [16]. For each protein structure, multiple sites of a unique ligand were analyzed for redundancy by comparing the counts of each residue. Binding MOAD uses biounit structures, which can contain multimeric proteins; in fact, 1958 of the 3295 complexes involved multimeric proteins. Biounits are the entire biologically relevant structure. Each multimeric structure was treated as “one entire protein” when identifying surface residues, and no buried residues in the interface were accidentally counted as exposed. Of course, some interface residues are solvent exposed, and any binding sites located between monomers were properly defined as being composed of exposed residues from both monomers during the surface area calculation. To avoid over-representing ligand sites of multimeric proteins, only one site was retained when multiple sites with an identical ligand and identical binding-site residues existed in the same structure. In multimers where the same ligand exhibited different contacts in symmetry-related pockets, one example of each case was included to represent the inherent variability of the binding. There were 2571 valid ligands (∼68% of the data set) represented by only one unique binding site in its respective protein biounit, while the other 1225 had more than one representation. For 923 ligands (∼24% of the data set), there were 2 non-redundant representations of the binding event. Among the remaining 8% of ligands in biounits, 171 (4.5%) had 3 unique binding sites, 80 (2.1%) had 4, and 1.4% had 5–14 unique representations. The valid ligands with more than 2 representations tended to be sugar molecules bound to sugar-processing enzymes. For invalid ligands, 717 out of 1485 ligands in biounits (∼49% of the data set) had a single representation in the respective biounit, 364 had 2, and the remaining 27% had 3 or more unique representations in a biounit. Dataset S1 in the supporting information lists the number of unique sites for each ligand in each biounit.

### Definition of Surface Residues

Solvent accessibility of residues was calculated using the NACCESS program [17]. NACCESS rolls a probe with the diameter of a water molecule across the entire van der Waals (vdw) surface of the protein and uses the path traced by the probe's center to calculate the SASA of each residue. It is important to note that this is different from the molecular surface area (MSA), which is the path traced by the probe's contact surface. Known ligands were removed from the structure before the SASA calculation. The default probe size was used, and any waters, hydrogens, or remaining HET groups were ignored (also default behavior). The NACCESS value of *abs_side* was used to define surface residues for the SC set and *abs_main* to define surface residues for the BB-only set. These report the absolute areas (in Å^2^) of the residue side chain, and backbone, respectively (calculated using default NACCESS atom types and vdw radii). Since NACCESS treats the Gly Cα as a side chain, the largest of the *abs_main* or *abs_side* values was used for that residue. SASA was calculated for all residues in a protein, which included any binding-site residues. We compared two definitions of “surface” residues: ≥5 Å^2^ SASA and ≥0.5 Å^2^ SASA.

### Residue Propensity Calculation

In accordance with previous studies, we used residue propensity as a measure of residue over-representation to explore the binding-site composition [1], [2], [4], [14]. The cumulative propensity *P_i_* for each amino acid *i = Ala, Arg, Cys …etc.* was calculated by taking the ratio of the frequency of the amino acid in binding sites *F_i_^BS^* and its frequency on the protein surface *F_i_^PS^*. The binding-sites frequency was obtained by summing across the surfaces of all binding sites *j = 1…J* in a binding-site class (SC or BB-only). The protein frequency *F_i_^PS^* was obtained by summing up the occurrence of the amino acid across the surfaces of all proteins *p = 1…P*, where *P* = 3295 in our case.

#### Equation 1: Propensity calculation


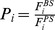
 where 
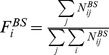
 and 
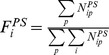


The propensities were calculated separately for valid versus invalid binding sites, and SC versus BB-only sets. Propensities greater than 1.0 show over-representation of a residue in the binding sites, relative to the entire protein surface, and values less than 1.0 show under-representation. Since propensity is a ratio of ratios and unit changes in its value represent fold changes in frequency, we present the propensity values on log-scaled axes.

Note that the residue counts were summed across the set of structures or binding sites before division. This is necessary because calculating a propensity value for a single protein may result in division-by-zero errors when rare residues, such as cysteine, are absent on the protein surface. Per-protein propensities for rare residues can also result in extremely large propensity values due to division by a small protein surface frequency, making summary results harder to interpret. Moreover, most binding sites do not contain all 20 common residues, which leads to many zero per-protein propensities. In calculations of propensities for a set of binding sites, only proteins that contained at least one site of that type (SC or BB-only, valid or invalid) were included in the calculations.

#### Hydrogen Bonding and van der Waals Contacts

As noted above, all interactions between the ligands and the residues were noted by their type (SC or BB-only, valid or invalid). The interactions were also noted at the atomic level to describe hydrogen-bonding and vdw interactions. All distances between ligands and the protein were calculated, and distances of 4.0 Å or less were tabulated. All interactions with distances greater than 3.5 Å and within 4.0 Å were counted as vdw interactions. Any distance of 3.5 Å or less were counted as vdw if they involved a carbon on either the protein or ligand. Distances between non-carbon atoms (N, O, S, P…) of the protein and ligand were counted as hydrogen bonds if they were within 3.5 Å. For tractability on this scale, angle criterion and more specific atom typing were not used to determine hydrogen bonding.

## Results/Discussion

Roughly a third the 3295 structures had invalid binding sites in addition to one or more valid site. The set yielded 7712 valid binding sites and 4909 invalid binding sites (Table 1), which together represent a comprehensive set of protein-ligand variety present in the PDB. However, it means that the number of binding-site residues available for frequency and propensity calculations is different between valid and invalid sites. After taking into account site redundancy and eliminating incredibly small binding sites (those that could not accommodate a solvent probe atom and thus did not have any “exposed” residues), there were 5562 valid and 3552 invalid sites. Again, Dataset S1 in the supporting information lists the number of unique sites for each valid and invalid ligand in each biounit.

**Table 1:** Summary data of structures from Binding MOAD used in the propensity calculations.

				SASA Cutoff = 5.0 Å^2^						SASA Cutoff = 0.5 Å^2^					
					Res/Site		SASA/Site (Å^2^)		Residues		Res/Site		SASA/Site (Å^2^)		Residues
Structures		Sites	Contact	NR Sites	Ave	Median	Ave	Median	Protein∶Site	NR Sites	Ave	Median	Ave	Median	Protein∶Site
**Valid**	3295	7712	SC	5562	11.4	11	433	399	10∶1	5514	13.2	12	441	406	10∶1
			BB-Only	3213	2.4	2	35	28	39∶1	3943	3.7	3	34	26	37∶1
**Invalid**	3295^a^	4909	SC	3552	3.6	3	194	178	50∶1	3581	4.1	4	195	178	51∶1
			BB-Only	1358	1.6	1	25	21	143∶1	1739	1.9	2	22	16	165∶1
**Only Enzymes** b	2354	6063	SC	4301	11.7	11	434	399	10∶1						
**Non-Enzymes** b	835	1597	SC	1261	10.3	10	431	401	11∶1						

The total number of binding sites and non-redundant sites are given. All SASA areas calculated by NACCESS. Enzyme class memberships determined based on EC annotations from the PDB.

a The same set of PDB structures were used to find valid and invalid binding sites.

b Data given for only the valid binding sites in enzymes and non-enzymes.

### “Raw” Contacts between the Proteins and Ligands

We first examined the raw contacts between all ligands and their binding sites, defined as all amino acids with at least one heavy atom (HA) within 4.0 Å of the ligand's HA. On average, the binding sites of valid ligands have 17.7 residues that provide 11.6 hydrogen bonds and 108 vdw contacts, but the binding sites of invalid ligands have only 5.2 residues that provide 10.4 hydrogen bonds and 80 vdw contacts. This is expected because valid ligands in our set tend to be larger and more buried than invalid ones. It is appropriate that the ratio of hydrogen bonds to vdw contacts is larger for invalids, which is consistent with the more hydrophilic set of molecules found in the invalid set.

**Figure 1:**
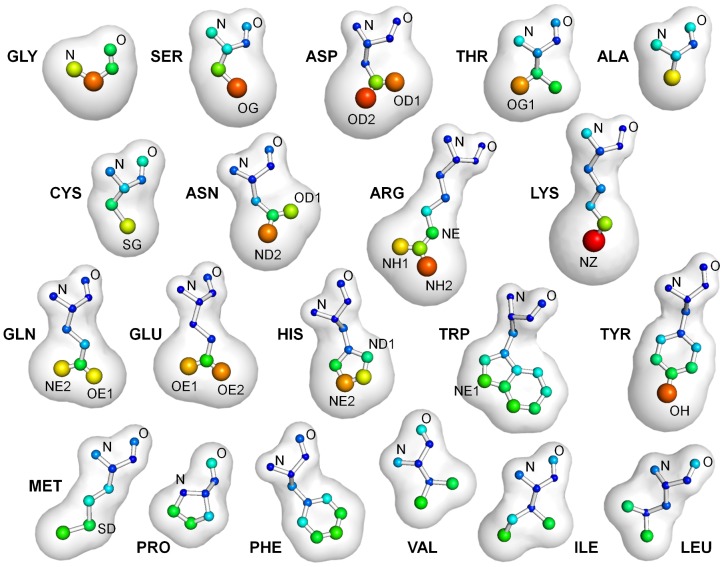
Bigger, hotter atoms have more “raw” contacts with ligands, on average. Each amino acid is shown with its total number of raw contacts represented by vdw radii and color. The average contacts per atom range 0.16 to 2.42, which has been offset and scaled to 1.0–3.0 vdw radii. The hotter colors indicate more contacts per atom: deep blue ≤0.30, cyan = 0.70, green = 1.00, yellow = 1.55, orange = 2.00, and red ≥2.30.

The total raw contacts for all residues interacting with valids are shown in Figure 1. The average number of interactions for each atom in each residue is denoted with increasing radii and hotter colors to represent more contacts. Clearly, the greatest interactions are to Gly and hydrogen-bonding side chains. Table 2 delineates the total raw contacts for both valid and invalid ligands, and Dataset S2 in the supporting information gives the contacts to the atomic detail seen in Figure 1. Almost all residues have more contacts to valid residues (an additional 0.7 contacts/residue for Asn up to an additional 2.81 for Tyr), but His shows no significant difference. Only Cys has more contacts to invalids (+1.4 contacts/residues). Residues have 4–9 contacts to valid ligands, with Ala, Val, Leu, and Pro having less than 4.5 contacts/residues and Tyr, Arg, and Trp having 7.5 or more contacts/residues. Obviously, the largest residues are capable of providing many more contacts than the smallest. To correct for the size difference, we also compared the contacts per HA of the residues, which ranged 0.53–1.32. Leu, Ile, Val, Phe, and Pro had the fewest contacts per HA (≤0.62), and Cys, Ala, Thr, Asp, Ser, and Gly had the most with ≥0.78 contacts/HA.

**Table 2:** Comparison of “raw” ligand contacts to “surface” ligand contacts.

	All Contacts to Residues within 4 Å								All Contacts to Residues within 4 Å and with ≥5 Å^2^ SASA							
	Valids			Invalids			Diff (V – I)		Valids			Invalids			Diff (V – I)	
		Contacts per			Contacts per		Contacts per			Contacts per			Contacts per		Contacts per	
Res	%Site	Res	Atom^a^	%Site	Res	Atom^a^	Res	Atom^a^	%Site	Res	Atom^a^	%Site	Res	Atom^a^	Res	Atom^a^
**Gly**	**11.1**	5.3	**1.3**	*8.1*	*4.0*	*1.0*	1.3	**0.32**	6.8	6.5	**1.6**	*5.0*	*4.1*	*1.0*	2.4	**0.60**
**Ser**	6.6	5.8	0.96	*6.0*	*4.5*	*0.74*	1.3	0.22	5.0	6.8	1.1	*5.3*	*4.7*	*0.78*	2.1	0.35
**Asp**	5.7	7.1	0.88	*5.1*	*4.7*	*0.58*	2.4	0.30	5.6	7.4	0.93	*5.6*	*4.7*	*0.58*	2.8	0.35
**Thr**	6.3	5.8	0.82	*5.6*	*4.6*	*0.66*	1.1	0.16	5.5	6.6	0.94	*5.1*	*4.7*	*0.67*	1.9	0.27
**Ala**	6.1	**4.1**	0.82	*4.9*	*3.1*	*0.62*	1.0	0.20	4.0	5.1	1.0	*3.4*	*3.3*	*0.65*	1.8	0.36
**Cys**	**1.7**	4.7	0.78	***1.6***	*6.1*	***1.0***	**−1.4**	**−0.23**	**1.0**	6.5	1.1	***1.3***	*7.9*	***1.3***	**−1.5**	**−0.24**
**Asn**	4.7	6.2	0.77	*5.3*	*5.5*	*0.68*	0.7	0.09	4.4	7.1	0.88	*5.9*	*6.2*	*0.77*	0.9	0.11
**Arg**	6.7	8.4	0.77	***11.2***	*6.1*	*0.55*	2.3	0.21	**10.1**	9.0	0.81	***14.6***	*6.2*	*0.56*	2.8	0.25
**Lys**	4.5	6.7	0.75	*6.1*	*4.6*	*0.51*	2.2	0.24	5.8	6.8	0.75	*7.9*	*4.4*	*0.49*	2.4	0.26
**Gln**	2.8	6.3	0.70	*3.4*	*4.4*	*0.49*	1.9	0.21	3.1	7.1	0.78	*4.0*	*4.5*	*0.50*	2.5	0.28
**Glu**	4.3	6.3	0.70	*5.5*	*4.5*	*0.50*	1.8	0.20	4.0	6.3	0.70	*6.2*	*4.5*	*0.50*	1.8	0.20
**His**	4.5	6.9	0.69	*6.0*	***7.2***	*0.72*	−0.3	−0.03	4.8	8.5	0.85	*6.5*	***8.3***	*0.83*	0.2	0.02
**Trp**	2.4	**9.1**	0.65	*2.3*	*6.9*	*0.49*	2.2	0.16	3.3	**10.8**	0.77	*2.6*	*8.0*	*0.57*	2.8	0.20
**Tyr**	5.7	7.6	0.64	*5.2*	*4.8*	*0.40*	**2.8**	0.23	7.2	9.0	0.75	*5.4*	*5.1*	*0.43*	**3.9**	0.32
**Met**	2.4	5.1	0.63	*1.7*	*3.6*	*0.45*	1.4	0.18	2.8	5.9	0.74	*1.9*	*4.0*	*0.50*	1.9	0.24
**Pro**	2.8	4.4	0.62	*3.8*	*3.1*	*0.44*	1.3	0.18	3.2	**4.7**	0.67	*4.2*	*3.2*	*0.45*	1.5	0.22
**Phe**	5.0	6.7	0.61	*4.5*	*4.2*	*0.38*	2.5	0.23	6.3	8.2	0.75	*3.9*	*5.0*	*0.45*	3.3	0.30
**Val**	5.2	4.3	0.61	*4.1*	*2.9*	*0.42*	1.3	0.19	4.7	4.8	0.68	*2.7*	***2.9***	*0.41*	1.9	0.27
**Ile**	5.0	4.7	0.58	*3.8*	*3.0*	*0.38*	1.6	0.20	5.3	5.6	0.70	*3.1*	*3.2*	*0.40*	2.3	0.29
**Leu**	6.4	4.3	**0.53**	*5.9*	***2.9***	***0.36***	1.4	0.17	7.1	4.8	**0.60**	*5.4*	*2.9*	***0.36***	1.9	0.24

Average contacts for valid and invalid ligands are compared across all residue types. The values and differences are given in both contacts/amino acid and contacts per non-hydrogen atom. The maximum and minimum values in each column are noted with bold; values for invalid ligands are noted in italics. Due to rounding, columns may occasionally sum to a value other than 100%.

a Number of non-Hydrogen atoms in each residue.

### Ligand Contacts with Protein Surface Residues

Of course, the raw contact information is interesting for understanding the molecular recognition of the ligands, but the raw contacts do not correct for the different frequencies of the amino acids in protein sequences. After all, more contacts with a particular residue is not significant if that residue is overwhelmingly present in the protein; random chance will result in increased contacts to that residue. Furthermore, we were concerned that many of the contacts were from residues that were not exposed on the protein surface. Typically, analyses of this sort concentrate on the exposed residues because correcting for the different frequencies of the amino acids is most appropriately done by comparing the surface of the binding site to the surface of the entire protein, which is discussed further below.

**Figure 2:**
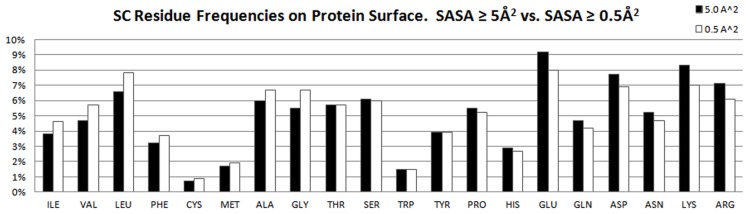
Frequencies of solvent-accessible SC with a cutoff of SASA ≥5 Å^2^ and SASA ≥0.5 Å^2^. Residues are sorted by decreasing hydrophobicity. With the smaller cutoff, the pattern shifts to more hydrophobic residues because poorly exposed, interior residues are able to meet the criteria with only a small patch of exposed surface.

We chose to use the common standard of ≥5 Å^2^ SASA as the definition of a “surface” residue [18], [19]. However, we were concerned that this definition included only 84% of SC binding-site residues, so we also examined the effect of lowering the minimum SASA cutoff to 0.5 Å^2^ to ensure we were not omitting significant parts of the binding site. Lowering the cutoff for the surface definition increased the total number of binding-site residues so that 98% of the residues within interaction distance of the ligand were considered “surface”. However, the respective increase in total binding-site SASA was only 0.2%, a contribution so small that it can be misleading to count those residues. Furthermore, the 0.5-Å^2^ definition led to inappropriate frequencies for amino acids on the surface of the protein (Figure 2). Specifically, more hydrophilic residues such as Arg, Asp, Lys, and Glu have the highest surface frequencies with the 5-Å^2^ cutoff (>7%), which is in line with other studies [20]. Although the relatively hydrophobic Leu had high frequencies with both definitions, it is not appropriate that counting many small-SASA contributions (at 0.5-Å^2^ cutoff) should make Leu more frequent (7.8%) than Arg (6.1%) or Lys (7%). Including the minimal contributions of small-SASA residues simply leads to erroneous conclusions when counting residue frequencies and propensities.

When examining the sites based on their exposed residues (5 Å^2^-SASA definition), valid binding sites were ∼5 times larger in terms of the number of residues and ∼2 times larger by surface area than invalid ones. The smaller number of contacts for invalids is consistent with the data in Table 1 that shows invalid sites have smaller SASA and fewer residues. Table 2 shows that the average number of contacts per residue increases for both valids and invalids when focusing on the surface residues, though the trend is smaller in the invalids. The increase in the average number of contacts is the result of excluding buried amino acids with few, weak vdw contacts to the ligands. For invalids, the contacts/residue are only 2.9–8.3 and the contacts/HA are 0.36–1.3. However, the valids have more complementarity in their sites as demonstrated by contacts/residue ranging 4.7–10.8 and the number of contacts/HA ranging 0.6–1.6. Many of the general patterns seen in the raw contacts to valid ligands are still seen when focusing on the surface residues, which increases confidence that the findings are robust. The amino acids with the lowest contacts/residue are still Pro, Val, Leu, and Ala (<5.5 contacts/residue), and Arg, Tyr, and Trp are still the residues with the most (≥9 contacts/residue). For the contacts per HA, the top residues are still Asp, Thr, Ala, Cys, Ser, and Gly (>0.9 contacts/HA), and four of the five lowest are the same. Leu, Pro, Val, Ile, and Glu have ≤0.7 contacts/HA. Glu replaces Pro in the bottom-5.

Table 3 details the hydrogen-bonding contacts of the surface residues to the valid and invalid ligands. Half of the residues have little difference in their hydrogen bonding to valids vs invalids. Cys has significantly more hydrogen bonds to valid ligands. The only residues that showed more hydrogen bonds to invalid ligands were Arg and Thr. Their most similar counterparts, Lys and Ser, showed no significant difference between hydrogen bonds to valids or invalids.

**Table 3:** Comparison of the average number of hydrogen-bonding contacts to surface residues.

	Ave Hydrogen Bonds to VALIDS (Res within 3.5 Å and ≥5 Å^2^ SASA)										Ave Hydrogen Bonds to INVALIDS (Res within 3.5 Å and ≥5 Å^2^ SASA)									
	Backbone		Side Chains						Sum H Bonds		Backbone		Side Chains						Sum H Bonds	
Res	N HB	O HB	Atom	HB	Atom	HB	Atom	HB	All	All/Atom^a^	N HB	O HB	Atom	HB	Atom	HB	Atom	HB	All	All/Atom^a^
**Cys**	0.18	0.08	**SG**	0.80					1.07	**0.36**	0.13	0.03	**SG**	0.35					0.51	**0.17**
**Gly**	0.39	0.28							0.68	**0.34**	0.41	0.22							0.62	**0.31**
**Ser**	0.22	0.11	**OG**	0.50					0.83	**0.28**	0.22	0.12	**OG**	0.41					0.75	**0.25**
**Asn**	0.14	0.11	**OD1**	0.37	**ND2**	0.45			1.08	**0.27**	0.11	0.11	**OD1**	0.28	**ND2**	0.36			0.85	**0.21**
**His**	0.07	0.09	**ND1**	0.29	**NE2**	0.47			0.90	**0.23**	0.07	0.06	**ND1**	0.30	**NE2**	0.32			0.74	**0.19**
**Arg**	0.07	0.05	**NE**	0.23	**NH1**	0.33	**NH2**	0.44	1.13	**0.23**	0.07	0.04	**NE**	0.29	**NH1**	0.46	**NH2**	0.61	1.47	**0.29**
**Thr**	0.12	0.11	**OG1**	0.42					0.66	**0.22**	0.23	0.11	**OG1**	0.46					0.79	**0.26**
**Asp**	0.10	0.12	**OD1**	0.33	**OD2**	0.30			0.84	**0.21**	0.14	0.13	**OD1**	0.20	**OD2**	0.20			0.66	**0.17**
**Gln**	0.10	0.10	**OE1**	0.30	**NE2**	0.31			0.81	**0.20**	0.14	0.08	**OE1**	0.18	**NE2**	0.30			0.71	**0.18**
**Tyr**	0.07	0.07	**OH**	0.42					0.56	**0.19**	0.05	0.07	**OH**	0.26					0.37	**0.12**
**Ala**	0.19	0.18							0.37	**0.19**	0.14	0.12							0.26	**0.13**
**Lys**	0.14	0.06	**NZ**	0.35					0.56	**0.19**	0.12	0.06	**NZ**	0.39					0.57	**0.19**
**Glu**	0.07	0.11	**OE1**	0.26	**OE2**	0.29			0.72	**0.18**	0.14	0.10	**OE1**	0.18	**OE2**	0.24			0.67	**0.17**
**Met**	0.09	0.09	**SD**	0.34					0.53	**0.18**	0.19	0.08	**SD**	0.11					0.38	**0.13**
**Trp**	0.05	0.07	**NE1**	0.26					0.38	**0.13**	0.04	0.05	**NE1**	0.31					0.40	**0.13**
**Pro**	0.10	0.12							0.23	**0.11**	0.08	0.11							0.19	**0.09**
**Val**	0.09	0.11							0.21	**0.10**	0.04	0.09							0.13	**0.06**
**Leu**	0.10	0.09							0.19	**0.10**	0.10	0.10							0.20	**0.10**
**Ile**	0.08	0.09							0.17	**0.09**	0.07	0.09							0.16	**0.08**
**Phe**	0.08	0.08							0.16	**0.08**	0.09	0.07							0.16	**0.08**

Hydrogen bonding of all valid and invalid ligands are compared across all residues that meet the surface definition. Both backbone and side-chain atoms are listed. The values and differences are given in both hydrogen bonds per residue and contacts per hydrogen-bonding atom. Due to rounding, columns may occasionally sum to a value other than 100%.

a Sum of all hydrogen bonds per number of hydrogen-bonding atoms.

### Residue Frequencies and Propensities

**Figure 3:**
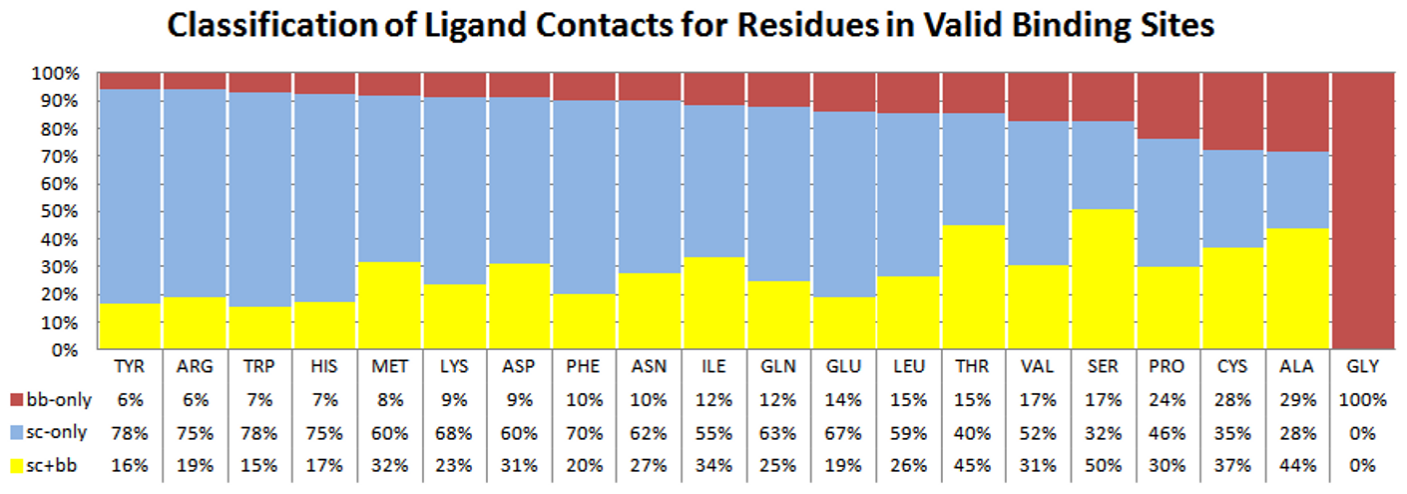
Relative frequency of SC-only, BB-only or both (SC+BB) interactions per residue. The residues with “SC” interactions in our analysis combine the SC-only and “SC+BB” contacts (blue+yellow). Residues are ordered by increasing BB-only frequency. Here, all Gly interactions are shown as BB-only to show its overall contribution to BB-only contacts. Due to rounding, columns may occasionally sum to a value other than 100%.

**Figure 4:**
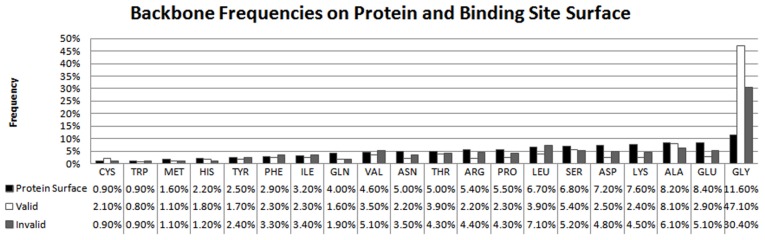
Frequencies of BB-only contacts in binding sites, sorted by increasing frequency on the protein surface. Surface residues with 5 Å^2^ or greater backbone SASA are shown. Gly interactions are shown as BB-only to stress that it constitutes the vast majority of such contacts. Due to rounding, rows may occasionally sum to a value other than 100%.

The BB-only interactions are relatively rare (Table 1) and are dominated by Gly (Figures 3 and 4). Gly provides over twice as many backbone interactions as any other amino acid; this is true for valids and invalids, raw and surface-residue contacts. Most residues with BB-only contacts to the ligand point their side chains away from the ligand, otherwise a side-chain atom would likely be within the interaction distance, and the residue would be classified as having SC contacts. Additionally, since BB-only contacts represent equivalent atom types from residue to residue, they are not expected to provide diverse interaction environments based on residue type. For all these reasons, we focus our discussion on residues in the SC category, which includes all Gly and residues with SC-only and SC+BB interactions.

Most proteins from the PDB exist in aqueous environments. Therefore, it is generally accepted that the solvated outer surface of the protein is composed of amino acids that tend to be hydrophilic in nature. Conversely, the core of the protein is more hydrophobic, a factor that contributes to the proper folding and stability of proteins [21], [22]. The opposite can be true for membrane bound domains, but soluble proteins have hydrophobic residues that tend to bury larger areas of their side chains upon protein folding than hydrophilic ones [23]. However, the composition of the solvent-exposed protein surface is not uniformly hydrophilic in nature, and the correlation between residue hydrophobicity and solvent-exposure is limited [20], [23]. Since binding sites are a part of a protein's surface, the comparative analysis of binding-site composition must be performed with respect to the composition of the entire protein surface.

**Figure 5:**
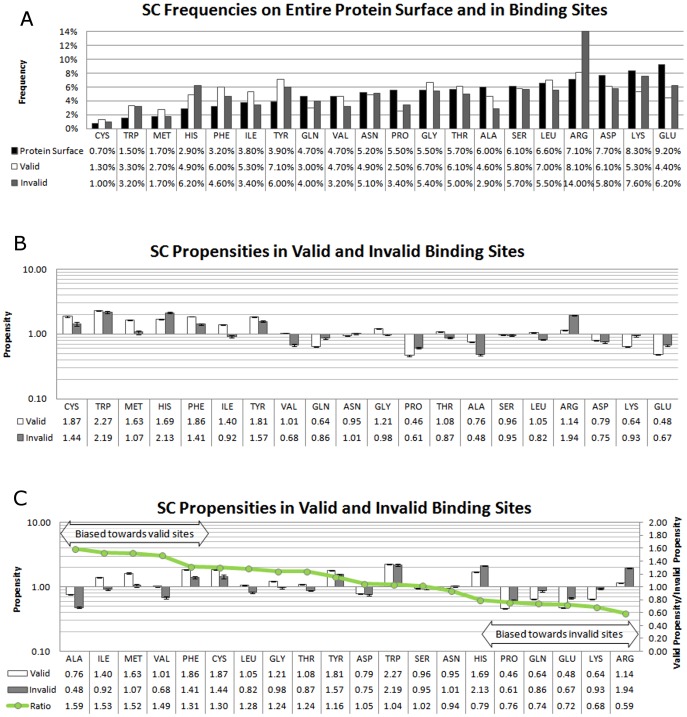
Frequencies and propensities of surface residues. A) Frequencies of solvent-accessible side chains on the protein surface and in binding sites with SASA cutoff ≥5 Å^2^. Due to rounding, rows in A) may occasionally sum to a value other than 100%. B) Median propensity of residues in ligand binding sites of valid and invalid ligands, analyzed across all proteins. Residues in A and B are ordered by increasing frequency on surface. C) Ratio of residue propensity for valid versus invalid binding sites. Residues ordered by decreasing ratio. Error bars in B and C indicate 95^th^ percentiles of 10,000 leave-10%-out samples.

In our analysis, charged and polar residues make up the largest portion of protein surfaces (black bars in Figure 5A), but surprisingly, Ala is more prevalent than the more hydrophilic Thr and similarily, Leu is more prevalent than and Ser. All four of these residues are frequent in sequence. Less-frequent hydrophobic residues such as Met, Phe, Trp, and Cys have low surface frequencies. If we relax the surface definition to include less-solvent-accessible residues, (Figure 2) very hydrophobic amino acids like Ile, Val, and Leu increase in their relative surface frequency. However, as discussed previously, their contribution in terms of fraction of overall surface area would be miniscule. Gly, which is common in protein sequences and unusual in its number of backbone interactions with ligands, has a surface frequency comparable to Asn and Pro.

Residue propensities in Figures 5B and 5C present the bias for residues to appear in protein surface regions involved in ligand binding. Pro, Glu, Gln, Lys, and Ala disfavor binding sites (propensities of 0.46–0.76). Arg, Thr, Val, Leu, Ser, and Asn have propensities within ±0.2 of 1.0, showing that these are relatively unbiased in their contributions to binding sites versus the rest of the protein surface (Figure 5B). Though Arg, Leu, and Asp have the first, third, and fourth largest contributions to binding sites (Figure 5A) their relative propensities are ∼1 because of their equally high prevalence on the entire protein surface. Larger propensities for binding sites occur when a residue is frequently observed in binding sites, but is rare on the general surface. Cys, Trp, Met, His, Phe, Ile, and Tyr all have low protein surface frequencies (left side of Figure 5A), and show propensities of ≥1.4 (left side of Figure 5B). Tyr and Phe are excellent examples. They are the second and seventh most common resides in binding sites, respectively, and they are rare on the protein surface. These residues are bulky and aromatic, so their exposure to solvent is rather unfavorable. It is reasonable that evolution is judicious in their use, placing them where they are most needed for a functional role, such as conservation in binding sites [2], [5], [20]. Trp also has a high propensity for binding sites, and similar physical properties, but its exceptional propensity actually reflects its rarity on the protein surface (<2% of all SC contacts). The same pattern is seen for Cys, which is even more rare on the surface (<1% of SC contacts). Gly is notable because backbones are uncommon on protein surfaces (about 17% of the total protein surface area), but when they are present, they are overwhelmingly Gly. Gly alone accounts for 13% of all backbone protein surface area (data not shown), and they tend to provide a large percentage of amino acids in binding sites. Gly backbones account for ∼50% of BB-only interactions in valid binding sites. However, when normalized relative to the whole protein surface, Gly shows a more modest propensity for binding-site regions (center of Figure 5B). Overall, our propensities for valid binding sites agree well with previously published propensities from a set of ∼35,000 redundant ligand-binding sites (R^2^ = 0.81 and Spearman ρ = 0.91 in comparison to Davis and Sali [24]), and those from a smaller set of 41 drug-binding sites (R^2^ = 0.79 and Spearman ρ = 0.79 in comparison to Soga *et al.* [14]). Propensities for invalid sites were less well correlated with these data (R^2^ = 0.27 and R^2^ = 0.61, respectively).

### Comparison of Frequencies and Propensities in Valid versus Invalid Sites

A unique aspect of this study is our ability to compare the binding-site interaction patterns for valid ligands to those in sites of spurious additives. This provides a type of “experimental control” which is usually not possible in analyses of binding-site databases. The issue at hand is not necessarily the recognition of additives themselves, but instead, with how valid and invalid binding differs. Figure 5C demonstrates the propensities for valid and invalid binding sites, ordered by the ratio between of the two. This data emphasizes our caution in over-interpreting the high propensities of Cys and Trp. They do not show any significant bias for valid ligands over invalids. One could argue that Trp, Cys, or any other residue may be inherently “sticky” for *all* small molecules, so of course, they will attract both valids and invalids. Who cares if there is a bias when these residues denote small-molecule binding sites? On the contrary, we find that there *are* residues which show a significant bias between the classes. This significance was confirmed by randomly shuffling valid and invalid “labels” 1000 times (maintaining their relative proportion) and re-calculating the propensities and ratios each time. All residues had an average ratio of 1 across the shuffled sets. The maximum and minimum of the shuffled ratios was 1.2 and 0.8 respectively, both for Cys, with all other residues having considerably narrower minimum and maximum values (data not shown). We therefore consider propensity ratios >1.2 and <0.8 as significant trends.

Ala, Ile, Met, and Val are the most biased toward biologically relevant binding sites over indiscriminant associations (ratio >1.4), followed by a second tier of Phe, Cys, Leu, Gly, and Thr (ratio >1.2). Conversely, His, Pro, Gln, Glu, Lys, and Arg show a bias towards invalid binding sites (ratio <0.8), although all but His and Arg have propensity for the surface rather than binding sites. Considering Arg has among the highest catalytic propensities [1], it should be present in many valid binding sites, but we do not see strong correlations between binding-site propensity (valid or invalid) and catalytic propensity (data not shown) or large differences in propensity values when enzymes are considered separately from non-enzymes (discussed further below). Instead, looking at the distribution of Arg interactions in binding sites (Tables 4 and 5) demonstrates that they make up most SC interactions in 11 of the top-20 ligand sites and are present at high rates (>15% of SC interactions) in sites of small, charged molecules, such as sulfate (ligand name of SO4 in the PDB), phosphate (PO4), acetate (ACY), and chloride (CL) ions. They are also especially frequent in citrate (CIT) sites, which appear on both valid and invalid lists, depending on the function of the bound protein. Of the residues that show valid to invalid ratios of >1.2, only Ile, Met, Phe, and Cys show a propensity for binding sites versus the protein surface.

**Table 4:** Composition of binding sites for the top-20 valid ligands.

HET	#Lig (%)	Ala%	Arg%	Asn%	Asp%	Cys%	Gln%	Glu%	Gly%	His%	Ile%	Leu%	Lys%	Met%	Phe%	Pro%	Ser%	Thr%	Trp%	Tyr%	Val%
**NAD**	250 (4.49)	5.90	4.75	6.12	7.01	1.56	2.14	3.43	7.49	3.60	**8.33**	6.82	4.01	1.87	4.58	4.51	5.98	7.94	1.44	4.80	7.73
**FAD**	217 (3.90)	6.77	7.09	4.02	4.47	1.91	3.51	4.28	7.33	4.72	6.79	6.21	3.98	1.42	4.65	4.00	6.91	**7.79**	3.42	6.28	4.44
**ADP**	172 (3.09)	4.48	10.46	5.43	5.37	0.50	2.35	3.36	**11.42**	2.80	4.98	5.20	9.96	1.85	4.14	2.74	5.76	8.67	0.78	5.04	4.70
**NAP**	165 (2.97)	6.37	8.97	5.68	4.02	0.59	2.25	2.01	**9.93**	3.01	6.78	5.99	5.64	2.01	2.25	4.12	8.76	8.65	0.97	5.78	6.23
**FMN**	130 (2.34)	5.09	**10.99**	7.17	2.43	1.39	3.88	2.14	9.14	5.73	4.34	4.63	4.51	3.18	3.30	2.89	8.21	6.94	3.18	6.54	4.34
**ATP**	100 (1.80)	2.76	**12.20**	4.26	6.27	0.17	2.42	7.52	10.78	2.26	4.43	5.51	12.03	1.92	5.43	0.75	5.35	8.02	1.42	2.26	4.26
**GDP**	96 (1.73)	3.17	4.39	3.98	11.44	2.96	1.74	3.17	8.27	1.43	1.94	8.27	**19.10**	0.31	4.60	1.63	8.17	10.52	–	1.94	2.96
**GLC**	86 (1.55)	3.95	9.65	6.58	**12.94**	0.22	6.14	7.46	3.07	7.46	2.41	1.10	2.63	2.19	7.46	0.88	1.75	1.54	11.40	10.53	0.66
**NDP**	76 (1.37)	6.19	9.32	4.66	3.83	1.18	2.30	2.85	9.32	2.85	5.29	5.85	5.78	2.64	1.32	2.85	**10.44**	8.35	1.74	7.38	5.85
**SAH**	67 (1.20)	5.07	2.97	3.21	10.51	1.85	2.10	4.45	**11.50**	2.35	5.07	8.16	1.98	4.45	7.91	2.97	6.06	3.83	4.20	7.29	4.08
**ANP**	61 (1.10)	4.90	7.48	6.62	7.23	–	3.43	4.04	**10.54**	1.84	6.37	4.78	9.56	2.21	4.53	1.84	5.51	7.97	0.98	4.29	5.88
**COA**	54 (0.97)	8.85	7.51	3.35	2.95	0.80	4.29	0.94	7.24	4.29	4.56	8.45	**8.98**	4.29	6.84	2.55	6.43	4.16	1.88	5.36	6.30
**NAG**	45 (0.81)	2.34	6.54	**19.16**	9.35	3.74	4.21	3.74	4.21	1.40	2.80	4.21	2.34	1.87	3.27	1.40	2.34	5.61	14.49	4.67	2.34
**CIT**	44 (0.79)	3.04	**16.22**	7.77	4.73	0.34	2.03	3.04	6.76	11.15	4.73	3.72	6.42	2.03	2.70	3.38	7.77	4.73	2.03	5.74	1.69
**AMP**	43 (0.77)	4.48	**10.70**	2.74	5.72	1.74	3.73	5.97	6.97	5.97	5.97	4.98	5.97	1.49	6.72	1.74	5.97	7.71	1.00	6.47	3.98
**NAI**	42 (0.76)	7.79	3.89	6.17	7.38	0.13	2.28	2.55	8.72	2.15	**9.40**	8.99	4.83	2.68	1.88	3.49	7.38	6.31	0.67	4.30	8.99
**MAN**	40 (0.72)	5.91	–	**18.72**	16.75	–	9.36	1.97	5.91	2.46	–	5.42	3.45	–	1.48	2.46	1.97	3.94	5.42	12.32	2.46
**SAM**	37 (0.67)	5.20	4.98	3.62	**11.09**	0.45	3.85	6.11	8.82	5.43	5.20	7.24	2.71	2.26	7.92	4.07	4.98	3.85	2.04	7.01	3.17
**GNP**	36 (0.65)	4.22	0.84	2.95	8.44	1.90	2.11	1.27	12.66	0.84	1.27	8.02	**18.78**	0.42	5.49	3.16	8.86	14.14	–	3.16	1.48

Ligand listed in decreasing fraction of 5562 binding sites. Most frequently interacting residue for each ligand is in bold. Due to rounding, rows may occasionally sum to a value other than 100%.

**Table 5:** Composition of binding sites for the top-20 invalid ligands.

HET	#Lig (%)	Ala%	Arg%	Asn%	Asp%	Cys%	Gln%	Glu%	Gly%	His%	Ile%	Leu%	Lys%	Met%	Phe%	Pro%	Ser%	Thr%	Trp%	Tyr%	Val%
**SO4**	903 (26.09)	2.22	**24.72**	4.85	4.02	0.69	4.05	5.37	4.88	8.03	1.35	2.74	10.42	1.28	1.84	2.98	7.24	5.89	1.87	4.05	1.52
**GOL**	586 (16.93)	3.03	**11.63**	4.95	7.53	0.31	4.55	7.89	5.17	4.55	4.10	5.35	7.35	1.29	4.95	3.39	4.95	4.68	3.88	6.68	3.79
**EDO**	343 (9.91)	3.37	**10.50**	6.50	6.35	0.78	5.09	6.50	4.23	3.68	5.25	6.58	6.74	1.18	5.88	4.08	4.23	4.86	4.47	6.58	3.13
**CL**	235 (6.79)	2.42	**15.80**	7.62	3.90	1.67	2.79	3.16	6.69	10.04	1.86	5.39	10.04	1.12	2.79	4.83	5.39	5.39	1.86	4.09	3.16
**PO4**	189 (5.46)	2.22	**18.03**	4.30	7.21	0.97	3.74	6.38	9.71	9.02	0.97	1.53	10.68	0.69	2.36	1.80	8.74	4.58	0.69	4.58	1.80
**ACT**	112 (3.24)	1.81	**13.18**	3.10	3.62	1.55	4.39	6.20	2.58	9.30	4.65	6.20	8.79	2.07	5.94	1.81	5.94	3.36	2.58	7.75	5.17
**MPD**	78 (2.25)	3.26	8.31	5.34	8.31	–	3.56	6.53	4.15	2.67	5.04	8.31	3.26	2.67	6.82	6.53	4.75	3.26	3.56	**9.50**	4.15
**EGL**	64 (1.85)	1.55	**11.92**	5.70	7.25	–	4.15	5.18	1.55	6.22	2.59	8.81	7.77	1.55	6.22	6.74	2.59	7.25	1.55	6.74	4.66
**FMT**	54 (1.56)	3.80	**13.92**	8.23	11.39	–	2.53	3.80	5.70	5.06	1.90	3.16	8.86	1.27	0.63	2.53	5.70	9.49	4.43	3.80	3.80
**TRS**	40 (1.16)	2.11	5.79	6.32	**9.47**	1.05	5.79	3.16	8.95	4.21	4.74	7.37	5.79	1.58	2.63	4.21	2.11	4.74	6.32	6.84	6.84
**ACY**	38 (1.10)	1.87	**18.69**	5.61	9.35	–	1.87	2.80	6.54	3.74	2.80	3.74	8.41	2.80	5.61	3.74	8.41	6.54	0.93	5.61	0.93
**PEG**	34 (0.98)	7.08	9.73	2.65	10.62	0.88	1.77	8.85	7.96	4.42	–	**12.39**	3.54	1.77	2.65	4.42	4.42	2.65	3.54	7.08	3.54
**IPA**	28 (0.81)	3.23	**11.83**	5.38	3.23	3.23	1.08	–	2.15	6.45	7.53	**11.83**	–	4.30	8.60	3.23	8.60	2.15	2.15	7.53	7.53
**BOG**	27 (0.78)	1.37	6.85	1.37	2.05	–	0.68	4.11	3.42	2.74	11.64	**19.86**	3.42	2.05	13.70	3.42	2.05	3.42	5.48	4.79	7.53
**IOD**	26 (0.75)	2.13	**12.77**	4.26	2.13	2.13	2.13	–	2.13	4.26	2.13	6.38	**12.77**	2.13	2.13	6.38	8.51	10.64	8.51	6.38	2.13
**EOH**	22 (0.64)	3.45	8.62	5.17	6.90	5.17	5.17	5.17	6.90	6.90	5.17	10.34	–	–	1.72	–	5.17	5.17	5.17	**12.07**	1.72
**BR**	20 (0.58)	–	6.98	6.98	–	–	6.98	6.98	4.65	6.98	9.30	2.33	4.65	2.33	9.30	**18.60**	–	2.33	–	6.98	4.65
**MES**	19 (0.55)	4.71	9.41	4.71	3.53	–	5.88	8.24	2.35	4.71	2.35	**10.59**	5.88	1.18	5.88	7.06	8.24	2.35	5.88	4.71	2.35
**MG**	18 (0.52)	–	10.00	6.67	**53.33**	–	3.33	20.00	–	3.33	–	–	–	–	–	–	–	3.33	–	–	–

Ligands listed in decreasing fraction of 3461 binding sites. Most frequently interacting residue for each ligand is in bold. Due to rounding, rows may occasionally sum to a value other than 100%.

In solution, all charged side chains may be expected to attract small, polar ligands classified as invalid in our dataset. However, we see higher frequencies for positively charged residues (Arg, Lys) than for negatively charged ones (Glu, Asp) in invalid binding sites. It is unusual that Glu and Asp are under-represented in invalid binding sites because positively charged ions are present in buffers just like negative ions. Asp and Glu are indeed frequent in Mg^+2^ sites, where they comprise 22 of 30 residues across 18 sites. However, the binding of positive ions is not observed often in our dataset; Mg^+2^, Na^+^, and Ca^+2^, and are 20^th^ and 23^rd^ and 26^th^ highest occurring invalid ligands by frequency, and together, they represent less than 0.8% of all invalid binding sites. This is in contrast to Cl^−^, I^−^, and Br^−^, which all make the top-20 list, and comprise ∼8% of invalid sites (Table 5). The higher desolvation cost of a positive ion – particularly a divalent one – might make such binding interactions less frequent and thus less likely to appear in protein crystal structures (outside of functional active sites, where they frequently appear as co-factors).

### Assessment of Ligand Bias on Propensity Values

**Figure 6:**
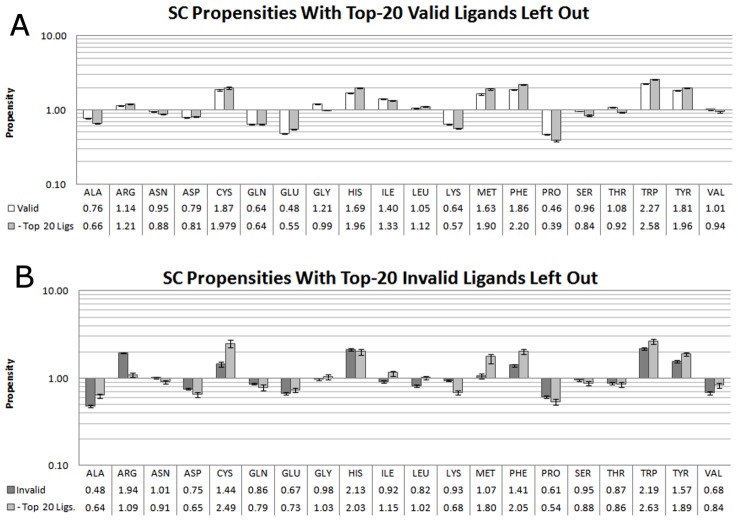
Propensities of SC interactions in valid sites, with and without the top-20 ligands by frequency. A) Propensities in valid sites. B) Propensities in invalid sites. The error bars represent 95^th^ percentile bounds based on leave-10%-out clustering within each set. Residues are ordered alphabetically.

There is a significant bias in the PDB among the valid ligands (abundance of nucleosides) and invalid ones (common buffer molecules). To measure the bias introduced by preponderance of such ligands, we recalculated propensities while leaving out any binding sites containing the most frequent 20 ligands given in Tables 4 and 5. Omission of the most frequent valid ligands (∼32% of the set) slightly raised propensities of Trp, Phe, His, Met, and Glu and lowered those of Ser, Ala, and Pro (Figure 6A). However, the omission had little effect overall. In contrast, propensities for invalid binding sites were significantly affected by the removal of the 20 most frequent invalids, which account for about 82% of invalid sites (Figure 6B). The propensities for Trp, Phe, Met, and Tyr rose sharply while propensities for Arg and Lys fell, indicating a respective increase and decrease in frequencies of these residues in the remaining binding sites (protein surface frequencies remained basically unchanged, data not shown).

These changes highlight the dependence of the propensities upon the size of the dataset and the variety of ligands it contains. While the propensities calculated for valid binding sites represent a broad array of ligands, invalid propensities are dominated by interactions that are made to the most frequent ligands, namely – sulfate, glycerol, ethylene glycol, and phosphate. This bias is inherent in protein crystallographic data and should be kept in mind when performing broad statistical analysis of residue interactions. Moreover, the large changes in propensities for the reduced set of invalid binding sites are hard to interpret, since subsets of such small size (352 structures remained) have large variation in the leave-10%-out cross-validation. In the next section, we examine how random subsets of such small size result in high standard deviations, even if all ligands are allowed. High standard deviations can indicate when an insufficient, small set of sites has been sampled. This exposes a caveat of any frequency- or propensity-based protein analysis with small sets of proteins: variation of binding-site frequencies in small sets of structures can have large effects on propensities (see below). Such comparison should only be done in the context of overall residue frequencies and with the knowledge of the uncertainty inherent to a small dataset.

### Influence of the Size of the Datasets on the Statistical Significance of the Propensities

**Figure 7:**
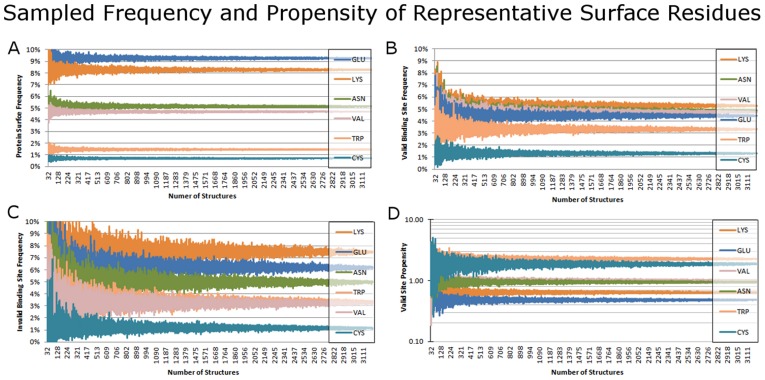
Examining the variation in the data, based on sample size. A) Protein surface, B) valid binding site, and C) invalid binding site frequencies, and D) valid binding site propensities of six residues. Values for subsets of the protein structure set, from 1% to 99% of the full set are shown, with 100 samples at each percent point.

To assess the statistical significance of the data, propensity calculations for each set of binding sites were carried out 10,000 times, each time leaving out a random 10% of the proteins (i.e., retaining ∼3000 structures at random). For each residue, the median of the 10,000 propensity values is reported, and the 95^th^ percentile bounds are used for the error bars. To assess the dependence upon the size of the dataset, a separate series of calculations were conducted using the procedure above. Progressively larger sets of proteins were randomly chosen from the set of 3295 structures, and propensities were calculated for that set without additional leave-10%-out sampling. The set size was incremented in intervals of 1% of the full structure set and 100 samples were taken at each percentage points, resulting in a total of 10,000 values. Frequencies and propensities were calculated for each sample (Figure 7). Additionally, propensity medians, standard deviation, and 95^th^ percentiles for six representative residues were calculated from 10,000 random samples at four different set sizes: 100, 500, 1000, and 2000 structures (Table 6).

**Table 6:** Median, standard deviation, and 95% confidence interval for the propensity of 6 representative residues.

Propensities			100 Structures		500 Structures		1000 Structures		2000 Structures	
*Residues with differing frequencies*			Valid	Invalid	Valid	Invalid	Valid	Invalid	Valid	Invalid
**Frequent**	**Lys**	97.5^th^ percentile	0.82	1.38	0.71	1.09	0.68	1.03	0.66	0.97
		**Median (st dev)**	**0.64 (0.08)**	**0.90 (0.21)**	**0.64 (0.03)**	**0.91 (0.08)**	**0.64 (0.02)**	**0.91 (0.06)**	**0.64 (0.01)**	**0.91 (0.03)**
		2.5^th^ percentile	0.48	0.55	0.57	0.75	0.60	0.81	0.62	0.86
	**Glu**	97.5^th^ percentile	0.62	1.01	0.54	0.80	0.52	0.76	0.50	0.71
		**Median (st dev)**	**0.48 (0.07)**	**0.66 (0.16)**	**0.48 (0.03)**	**0.66 (0.07)**	**0.48 (0.02)**	**0.67 (0.04)**	**0.48 (0.01)**	**0.67 (0.02)**
		2.5^th^ percentile	0.34	0.38	0.42	0.54	0.44	0.58	0.46	0.62
**Moderate**	**Val**	97.5^th^ percentile	1.30	1.19	1.12	0.88	1.08	0.81	1.05	0.75
		**Median (st dev)**	**1.01 (0.14)**	**0.66 (0.24)**	**1.01 (0.06)**	**0.68 (0.10)**	**1.01 (0.04)**	**0.68 (0.06)**	**1.01 (0.02)**	**0.68 (0.03)**
		2.5^th^ percentile	0.75	0.26	0.90	0.49	0.94	0.55	0.97	0.61
	**Asn**	97.5^th^ percentile	1.22	1.57	1.05	1.22	1.01	1.13	0.98	1.06
		**Median (st dev)**	**0.94 (0.13)**	**0.96 (0.26)**	**0.95 (0.05)**	**0.99 (0.11)**	**0.95 (0.03)**	**0.99 (0.07)**	**0.95 (0.02)**	**1.00 (0.04)**
		2.5^th^ percentile	0.71	0.52	0.85	0.79	0.88	0.86	0.91	0.92
**Rare**	**Cys**	97.5^th^ percentile	3.00	4.52	2.29	2.71	2.15	2.29	2.01	1.97
		**Median (st dev)**	**1.87 (0.52)**	**1.46 (1.13)**	**1.86 (0.21)**	**1.62 (0.47)**	**1.88 (0.14)**	**1.64 (0.30)**	**1.88 (0.07)**	**1.67 (0.16)**
		2.5^th^ percentile	0.96	0.00	1.48	0.89	1.61	1.13	1.73	1.35
	**Trp**	97.5^th^ percentile	3.13	4.06	2.60	2.98	2.49	2.72	2.38	2.52
		**Median (st dev)**	**2.25 (0.41)**	**2.20 (0.84)**	**2.28 (0.16)**	**2.29 (0.34)**	**2.27 (0.11)**	**2.29 (0.22)**	**2.27 (0.06)**	**2.28 (0.12)**
		2.5^th^ percentile	1.53	0.77	1.96	1.64	2.07	1.87	2.16	2.06

The values are given to show the importance of using a large dataset and the variation possible when using small subsets of protein structures. All values based on 10,000 random samples from the full protein set. The confidence interval ranges from the 2.5 percentile to the 97.5 percentile of the distributions from those samples.

The variation in SC frequencies and propensities were thus assessed by sampling random sets of varying numbers of structures (Figure 7) 100 times each. For clarity we focused on 6 representative residues: Lys and Glu as the most frequent on protein surface, Val and Asn as moderately frequent, and Cys and Trp as the least frequent. The protein surface contains the most residues by number, and the residue frequencies converge to within ±0.5% variation once ∼500 or more structures are sampled (Figure 7A). The binding sites are much smaller than the protein surface, so a larger number of structures are needed to achieve convergence of ±0.5% variation: ∼1500 structures for valid sites (Figure 7B) and ∼2500 structures for invalid sites (Figure 7C). The propensity values fluctuate in proportion to the frequencies (Figure 7D) and converge around ∼1000 structures in a dataset. Standard deviations of propensities for Lys and Glu in valid and invalid binding sites are below 0.1, even in subsets as small as 500 structures (Table 6). The propensities of rare residues do not converge to such small standard deviation until sets as large as 2000 structures are sampled, especially in the case of propensities for invalid sites. Convergence to mean values of the underlying population is guaranteed as the sample set size approaches the size of the full set; however, the rate of this convergence indicates whether relatively small subsets sufficiently sample the full population means. When constructing a dataset for computing propensities, a balance is required between eliminating redundant or poor quality structures and maintaining a sufficient set size. *Based on our results, a set of at least 1000 structures is required to confidently measure general binding-site propensities for valid ligands and 2500 are required for invalid ligands.* Of course, these numbers are based on a random and non-redundant protein set. Frequencies and propensities for a set of related proteins (for example, those from the same structural fold family) may show such convergence with fewer structures. We recommend that any propensities calculated on a limited set of structures should be assessed by comparison to the best-available general propensities (such as ones presented here) and by taking into account the variation in random subsets of similar size.

**Figure 8:**
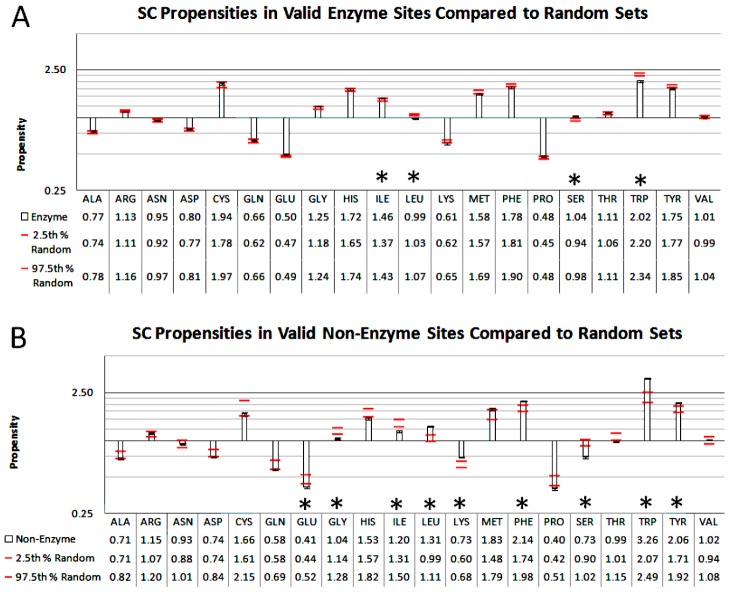
Propensities in valid binding sites. Propensities are broken down into A) enzyme and B) non-enzyme proteins. The black error bars represent 95^th^ percentile bounds based on leave-10%-out clustering. For context, red lines represent 95^th^ percentile bounds of propensities from 10,000 random samples of A) 2500 random, diverse proteins and B) 1000 random, diverse proteins (as seen in Table 4). Stars indicate residues whose median propensity value (leave-10%-out 95^th^ percentile error) falls outside of the 95^th^ percentiles of the randomly-sampled propensities.

As an example, we looked at the differences in propensities between enzyme and non-enzyme, valid-ligand binding sites, which have been previously shown to differ in their ligand efficiencies [25]. Figure 8 shows the propensities along with red lines indicating the 95^th^ percentile bounds of valid propensities from random sets of structures sampled 10,000 times from the full dataset (as presented in Table 6). For enzymes, sets of 2500 structures were sampled, while for the smaller non-enzyme set only 1000 structures were sampled. The leave-10%-out sampling used during the propensity calculations provides a measure of stability for the propensity values. In contrast, the sampling of random structures provides a bound for propensity values that can be expected by chance. Therefore, for enzyme or non-enzyme propensities to be considered different from the general (randomly observed) valid binding-site propensities, their 95^th^ percentile range must be outside the 95^th^ percentile range of propensities obtained from random structure sets of the same size. The asterisks in Figure 8 mark residues that fulfill this criterion. This is the strictest-possible criterion, because only minimal overlaps of the median distributions can still be considered statistically significant. The average values of random sampling will be enzyme-biased because Binding MOAD and the PDB are themselves enzyme-biased. Therefore, exceptional propensity trends for non-enzyme may be more likely.

The set of enzyme structures makes up more than two-thirds of the structure set used to compute propensities in this study. Binding-site propensities computed on this number of structures are very close to general propensity trends seen across all valid binding sites. Accordingly, the variation of propensities in corresponding random samples is very low. In enzyme binding sites, Ile and Ser have median propensities higher than random, and Leu and Trp lower ones. The set of non-enzymes has nine residues that have propensities significantly different than those seen at random. Leu, Lys, Phe, Trp, and Tyr have significantly higher binding-site propensities than those seen in sets of random structures, and Glu, Gly, Ile, and Ser have lower-than-random propensities. In our recent study comparing residue composition of enzyme and non-enzyme sites, Leu, Met, Trp and Tyr were found to have much higher frequencies in binding sites of high-affinity, non-enzyme proteins than in enzyme, high-affinity binding sites [25]. Combined with our propensity observations, the presence of Leu, Trp, and Tyr residues in binding sites without enzymatic function may be a distinguishing trend for allosteric or regulatory sites. Although Met propensity is higher in non-enzyme sites, it is within random sampling error. Our previous study also observed relatively low non-enzyme binding-site frequencies for Val, Ile, Asp, and Gly. Our propensities for Ile and Gly are consistent with their findings, but Asp has no propensity trend among enzymes versus non-enzymes, aside from its low propensity for binding sites in general. The elevated propensity of Lys and Phe and lower propensities for Glu and Ser for non-enzyme sites are unique trends observed in the current study.

As smaller sets of structures are used for calculating propensity values, there is a greater chance of seeing values that deviate from general binding-site propensity trends. However, the 95^th^ percentile margins of error from randomly sampled sets of similar size will also change, becoming wider, especially for less-frequent residues. Therefore, it is important to conduct comparisons to randomly-sampled propensity values as suggested herein, to distinguish set-specific trends from the overall propensity trends in the currently available data.

### Conclusions

Our study highlights the differences in amino-acid interactions with valid and invalid ligands and the frequency of residues taking part in these interactions, in contrast to the surface composition of the whole protein. Most importantly, the relative propensity of valid versus invalid binding sites should help improve methods for identifying binding sites in proteins of unknown functions and improve other proteomic methods where understanding of general composition of protein-ligand binding sites is required.

Our data could have its greatest utility in scoring predicted sites. Most scores are based on a weighed sum of the presence of each amino acid. Typically, Trp is heavily weighted because of its high propensity (2.27), but it is possible that the weight should be more modest because the ratio of valid to invalid propensities is near 1. More importantly, His has a high propensity (1.69) which would call for a high weight, but we find it is biased for invalid sites. Given this, it is probably not appropriate to highly weight the presence of His residues in a score. Conversely, Ala's low propensity (0.76) would usually result in some sort of penalty to a score, but its 1.59 ratio of valid to invalid propensities shows that it is more biased to valid sites and likely does not deserve to be a penalty. Of course, the residues with high propensities and high valid/invalid ratios should be the best indicators and given the highest weights: Ile, Met, Phe, Cys. Better understanding of these interactions, and how they differ across binding sites, can help focus statistical analysis across broad sets of protein surfaces toward the most biologically relevant ligand sites.

Looking at the variation of shapes, sizes, and composition of protein-ligand binding sites and the ligands they bind, it is easy to see why finding a general method for predicting their location and binding partners is such a challenge. Recent studies of thousands of human protein-ligand complexes found a complicated relationship between the similarity of protein sequences and the similarity of their pockets and bound ligands [1], [12]–[14], making it difficult to predict novel valid binding sites by sequences. Assessing the shape and sequence-independent residue composition of a ligand site has emerged as an orthogonal way to identify valid binding sites on protein surfaces [12]–[14]. In a more direct illustration of the complementarity of the propensity data with other prediction approaches, a study by Soga et al. [14] examined pockets identified by a geometry-based prediction tool and a rank-score for binding sites based on a protein-ligand binding index. That index was similar to residue propensity and showed some clear success in finding known binding sites in a set of crystal structures. Our study offers atomic contacts and propensity values based on a higher quality, larger, and more diverse dataset to fuel similar efforts.

This study also exposes the variation in residue frequencies on the protein and binding-site surfaces, depending on the number of proteins. Given how this variation can affect the interpretation of frequency- and propensity-based analysis of protein surfaces, we recommend that at least 1000 diverse protein complexes are needed for significant general conclusions for biologically relevant valid binding sites. When calculating propensities for smaller sets of structures, such as proteins of a functional family or similar ligand-binding sites, it is important to compare them to those of randomly sampled sets of structures. This can help determine how significant the trends are with respect to the variety of protein-ligand sites currently available in databases such as Binding MOAD.

## Supporting Information

Dataset S1 **The number of unique binding events is given for every PDB file in the “count” column.** All ligands are named for each biounit file from the PDB. The valid ligands are listed on one page, and the invalid ligands are described on the other page.
(XLSX)

Dataset S2 **Average contacts between the ligands and the proteins.** Total count, hydrogen-bonding, and vdw contacts are detailed for both valid and invalid ligands. “Raw” contacts are given on one page, and contacts to surface residues (5 Å^2^-SASA definition) is given on the other page.
(XLSX)
